# Research on interdisciplinary measurement and evolutionary path in the field of ophthalmology

**DOI:** 10.3389/fopht.2025.1578956

**Published:** 2025-06-06

**Authors:** Lu Chunji, Liu Yuwei, Zhang Fangyuan, Nie Zihan

**Affiliations:** ^1^ Beijing Tongren Hospital, Capital Medical University, Beijing, China; ^2^ Institute of Medical Information, Chinese Academy of Medical Sciences, Beijing, China

**Keywords:** neurosciences, surgery, multidisciplinary sciences, biochemistry & molecular biology, psychology, medicine: genetics & heredity, cell biology, genetics & heredity

## Abstract

**Background:**

Interdisciplinary collaboration is an inevitable trend in solving complex problems. As medical research deepens, the integration of theories, methods, and technologies from other disciplines with medicine has become an effective means to promote the development of medical research and address issues in the medical field

**Objective:**

There are significant differences in disciplinary intersections in different subfields of medicine, and this study, using ophthalmology as an example, aims to tap into the complex interrelationships between ophthalmology and other disciplines, to reveal interdisciplinary themes in ophthalmology, and to track the development and evolution of trans-ophthalmologic research

**Methods:**

This article takes ophthalmology as an example and uses the Rao-Stirling index to calculate the disciplinary distribution of journals cited in the literature of this field. By setting a threshold, it identifies interdisciplinary literature. Based on the LDA model, it conducts topic identification for the interdisciplinary literature to discover the interdisciplinary themes. Furthermore, it analyzes the evolution trends of these themes using the principle of similarity and visualizes the evolutionary paths.

**Results:**

The paper successfully linked 85,401 pieces of literature with WOS subject classifications, involving a total of 250 WOS subjects. The top 10 cited subjects are: Ophthalmology, Neurosciences, Surgery, Multidisciplinary Sciences, Biochemistry & Molecular Biology, Psychology, Medicine: Genetics & Heredity, Cell Biology, Genetics & Heredity, Clinical Neurology. A total of 18,573 pieces of literature with a Rao-Stirling index greater than 0.7 were selected as high interdisciplinary literature in Ophthalmology. The literature was divided into time slices of two years each, and scores for thematic coherence and thematic perplexity were calculated. Ultimately, twelve themes were identified for 2014-2015, eleven themes for 2016-2017, eleven themes for 2018-2019, ten themes for 2020-2021, and nine themes for 2022-2023. Based on the calculation of thematic similarity, three main evolutionary paths were summarized. The evolutionary paths are: “1-1, 1-2, 1-3, 1-5, 1-6, 1-9,1-10→2-1, 2-4, 2-7, 2-8, 2-9, 2-10→3-4, 3-8, 3-10→4-1, 4-5, 4-8, 4-10→5-5, 5-8, 5-9”, “1-1, 1-2, 1-3, 1-5, 1-6, 1-9,1-10→2-1, 2-4, 2-7, 2-8, 2-9, 2-10→3-4, 3-8, 3-10→4-1, 4-5, 4-8, 4-10→5-5, 5-8, 5-9”

**Conclusions:**

This study conducts a comprehensive analysis of interdisciplinary themes in ophthalmology through the application of the Rao-Stirling index and the LDA model, revealing trends of multidisciplinary collaboration and evolving topics. To facilitate the integration of these themes, the research proposes several recommendations: first, to promote interdisciplinary collaboration in order to establish an innovative ecosystem that spans various fields; second, to develop a systematic funding framework for research and enhance the evaluation system for interdisciplinary projects; third, to implement reforms in ophthalmic education aimed at strengthening the cultivation of interdisciplinary talent; and fourth, to expand the scope of academic journal inclusions by establishing dedicated sections for interdisciplinary research.

## 1 Introduction

As societal advancements continue, traditional academic disciplines increasingly struggle to address numerous significant scientific challenges. Consequently, the dissolution of disciplinary boundaries and the promotion of knowledge cross-fertilization have emerged as essential trends in the evolution of academic fields. Interdisciplinarity refers to the interaction among two or more distinct academic disciplines, encompassing the exchange of ideas, the organization of concepts and methodologies, the integration of research and educational practices, and the broader incorporation of diverse epistemologies, terminologies, and data (1). In recent years, high priority has been given to interdisciplinarity at home and abroad. In 2015, the journal Nature published a series of feature articles that examined interdisciplinary discourse within the scientific community. Subsequently, in 2019, the National Natural Science Foundation of China (NSFC) introduced a funding guideline that emphasized a focus on commonalities and cross-disciplinary collaboration. Moreover, in 2020, the NSFC established the Department of Interdisciplinary Science with the aim of actively fostering interdisciplinary research initiatives.

Currently, there is a significant proliferation of research outcomes in the field of interdisciplinary studies. Scholars both domestically and internationally have primarily focused their investigations on two key areas: first, the measurement of interdisciplinary collaboration, including the design and application of relevant indicators, methods, and models. Depending on the measurement object, interdisciplinary measurement can be categorized into several types: author-centric measurement (2), literature-centric measurement (3), keyword-centric measurement (4), fund-centric measurement (5), and citation-centric measurement (6–8). Among them, measuring disciplinary diversity through literature citations is the most dominant measure, including the first generation of diversity measures dominated by Shannon entropy and Gini-Simpson diversity, the second generation of diversity measures dominated by Ricota-Szeidl entropy, Rao quadratic entropy and Rao-Stirling, and the third generation of diversity measures dominated by Hill’s numbers, Leinster-Cob-bold diversity metrics as the main third generation diversity measure. The Rao-Stirling index is the most prevalent due to its effective balance of variety, balance, and disparity (9). The second area of focus involves the identification of cross-disciplinary themes and the prediction of subject intersections. Deerwester S introduced Latent Semantic Indexing (LSI), which derives three sub-matrices that represent the relationships among words, topics, and documents through singular value decomposition of a matrix modeled by the TF-IDF algorithm (10). Hofmann T further developed a probabilistic Latent Semantic Indexing (pLSI) model, which conceptualizes each word in a document as a sample from a mixture model based on the generative probabilities of LSI (11). Additionally, Blei DM proposed the Latent Dirichlet Allocation (LDA) model, a more comprehensive probabilistic generative model that operates under the bag-of-words assumption and Devenet’s theorem, constructing a three-tiered hierarchical Bayesian generative model comprising documents, topics, and words (12). Wei X et al. noted that the LDA model surpasses traditional clustering-based methods, leading numerous researchers to adopt it for the subject recognition of scientific documents (13).

There is a clear trend of disciplinary crossover in the field of medicine, and the theoretical techniques of science and technology, engineering, social sciences, humanities and other disciplines have contributed greatly to solving problems in medical sciences (14), and studies have been conducted to examine the picture of disciplinary crossover associations in all fields of medicine using either qualitative or quantitative methods (15, 16), but disciplinary crossover varies significantly in different subfields of medicine. In this study, we taking ophthalmology as an example, the Rao-Stirling index was utilized to calculate the disciplinary distribution of journals to which the literature citations in this subject area belonged, and the disciplinary crossover literature was discovered by setting a threshold value. And then, based on the LDA model, the ophthalmology disciplinary crossover literature is subject identified, so as to discover the disciplinary crossover themes in this field. Finally, the similarity principle is used to produce Sankey diagrams to analyze the ophthalmology subject evolution path. The aim of this methodology is to clarify the complex interrelations among disciplines, uncover interdisciplinary themes in ophthalmology, and track the development and evolution of interdisciplinary research. The findings of this study contribute to a deeper understanding of the interdisciplinary framework within ophthalmology, thereby facilitating the advancement of the field.

## 2 Materials and methods

### 2.1 Materials

The previous ten years have represented a significant era of profound integration between ophthalmology and various fields, including artificial intelligence, gene editing, and materials science, etc. This investigation utilized articles categorized under ophthalmology from the SCI-EXPANDED database within the Web of Science as the primary research focus. The search strategy implemented was defined as Web of Science Category = (OPHTHAMOLOGY), with a publication date constraint set between 2014 and 2023. Specifically, the study concentrated on articles classified as “article” and published in the English language. The data retrieval was executed on March 15, 2024, yielding a total of 85,401 documents. To enhance the integrity of the dataset, the bibliographic information obtained was meticulously cleaned, resulting in the exclusion of incomplete entries, including abstracts, keywords, and references. As a result, a refined dataset comprising 82,841 articles was established, which included a cumulative total of 2,693,052 reference records.

### 2.2 Methods

**Figure 1:**
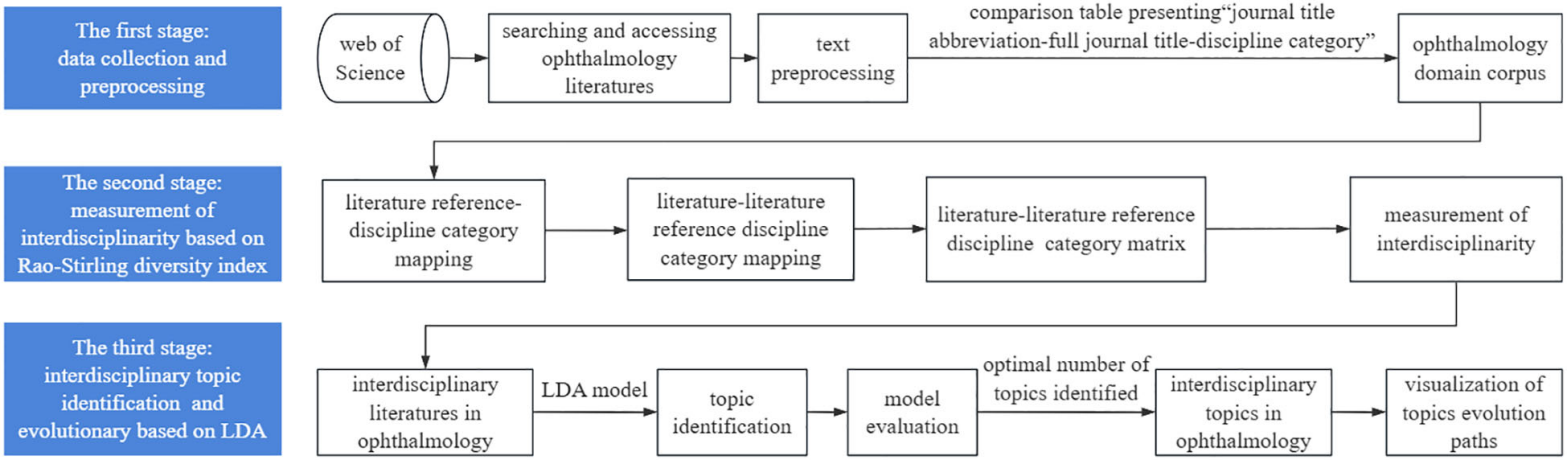
Technology roadmap.

In the present study, we utilized the Rao-Stirling index to examine the distribution of journals that include citations from ophthalmology literature. This approach allowed us to quantify the extent of interdisciplinary engagement within the literature, enabling the identification of instances of interdisciplinary research in the field of ophthalmology based on a predetermined threshold value. Additionally, we applied the LDA model to classify the subject matter of interdisciplinary literature, which aided in pinpointing specific interdisciplinary topics and visualizing the trajectories of topic evolution. The technology roadmap is illustrated in **Figure 1**. The specifications pertaining to the hardware and software utilized in data computing are delineated as follows: The hardware configuration comprises 32GB of 1600MHz DDR3 random access memory, a 2.2GHz Intel Core i7 central processing unit, and a 500GB hard disk serving as the storage medium. The software environment is characterized by a 64-bit Windows operating system, alongside the inclusion of various tools and programming languages such as Visual Basic for Applications (VBA), R, and Python, which are employed for data processing and analysis.

#### 2.2.1 Measurement of Interdisciplinarity based on Rao-Stirling diversity index

The Rao-Stirling index can not only measure the size level of disciplines, but also assess the structural characteristics of the distribution of disciplines, so as to comprehensively characterize the overall situation of disciplinary layout. In general, a higher Rao-Stirling index value indicates a greater diversity among disciplines, which also suggests a more pronounced degree of interdisciplinary intersectionality. The formula for calculating the Rao-Stirling diversity index is as follows (17, 18):


$$\text{RS}=\sum_{ij(i\neq j)}d_{ij}P_{i}P_{j}$$

In this study, the inter-disciplinary distance, represented as *d_ij_*, is quantified through the application of cosine similarity. The proportions of publications within specific disciplinary categories, indicated as *P_i_* and *P_j_*, are determined in relation to the aggregate number of publications across all disciplines.

#### 2.2.2 Identification of interdisciplinary topics and evolutionary modeling utilizing LDA

##### 2.2.2.1 Identification of interdisciplinary topics

LDA models usually use topic perplexity and topic coherence to determine the optimal number of topics (19, 20). In this study, a combined approach utilizing both topic perplexity and topic coherence is employed to ascertain the optimal number of LDA topics (21). Topic perplexity serves as a crucial indicator of the model’s ability to distinguish between topics, thereby assessing its generalizability to new data and accuracy in topic classification (22). Lower perplexity values indicate a stable topic structure with minimal expected error. Topic coherence, which reflects the semantic consistency among words within a topic, plays a pivotal role in determining the number of topics by indicating the strength of semantic relationships within a theme. Higher coherence values indicate greater semantic cohesion, which enhances the interpretability of the topic model.

The perplexity specific formula is as follows (23):


$$perplexity(D)=\text{exp}(-\frac{\sum_{m=1}^{M}\log p(W_{m})}{\sum_{m=1}^{M}N_{m}})$$

The symbol *Perplexity(D)* signifies the perplexity of the model, where $N_{m}$ refers to the number of words in the mth document within the test set, and *M* represents the total number of documents in the test set. Additionally, *p* $\left(W_{m}\right)$ indicates the probability distribution of words in the mth document.

The coherence specific formula is as follows (24):


$$coherence(V)=\sum v_{i},v_{j\in }V^{score(vi,\;vj,\epsilon )}$$

$\text{score}(v_{i,}v_{j})$ formula is as follows:


$$\text{score}(v_{i,}v_{j})=\log\frac{D(v_{i},v_{j})+1}{D(v_{j})}$$

Where V is a set of words belonging under the topic, ϵ  is the smoothing coefficient, $D(v_{i},v_{j})$ count the number of documents that contain the words $\;v_{i,}v_{j}$ , $D(v_{j})$ count the number of documents containing the word $v_{j}$ .

### 2.2.2.2 Analysis of the evolution of topics

The topics derived from the analysis of documents across various temporal intervals utilizing the LDA model may exhibit both similarities and distinctions. To elucidate the relationships of similarity and evolution among document topics in consecutive time periods, this study employs the calculation of cosine distance values to assess the evolutionary dynamics of these topics. The cosine distance is quantified by the cosine of the angle formed between two vectors in a vector space, with the following formula for its computation (25):


$$\cos\theta =\frac{\sum_{i=1}^{n}(x_{i}*y_{i})}{\sqrt{\sum_{i=1}^{n}{\left(x_{i}\right)}^{2}}*\sqrt{\sum_{i=1}^{n}{\left(y_{i}\right)}^{2}}}$$

Cosine distance values span from 0 to 1, with higher values indicating greater similarity between two vectors. By employing the LDA model to generate a set of topic words, a topic space vector is established. This vector facilitates the computation of similarity between two topics, thereby enabling the assessment of the extent of topic evolution.

## 3 Discussion

### 3.1 Distribution of citations in ophthalmology literature across disciplinary journals

The journal title abbreviations were extracted from the references and matched with the Web of Science Category of the respective journals in which they were published, and a comparison table was constructed for “Journal Title Abbreviation - Full Journal Title - Discipline Category”. A total of 2,293,869 reference records were successfully linked to their corresponding subject categories (**Table 1**). Among them, the document ID represents the document number of ophthalmology, the journal abbreviation, the full name of the journal, and the Web of Science category represent the correspondence of each reference in ophthalmology. When a journal reference was associated with multiple WOS subject categories, all categories were treated as equally important without assigning weights. This approach was chosen to avoid introducing bias and to allow for a more neutral and comprehensive representation of the journal’s disciplinary scope. The successful matches encompassed 85.2% of the total references, with an average of 1.27 subject categories aligning with each journal to which a reference was attributed.

**Table 1:** Disciplinary categories to which literature citations in the field of ophthalmology belong (part).

ID	References	Journal Title Abbreviation	Full Journal Title	WOS categories
1	Ang AY, 2013, CORNEA, V32, P229, DOI 10.1097/ICO.0b013e318255eac4 (26)	CORNEA	CORNEA	Ophthalmology
1	Baradaran-Rafii A, 2013, CORNEA, V32, P561, DOI 10.1097/ICO.0b013e31826215eb (27)	CORNEA	CORNEA	Ophthalmology
1	Biber JM, 2011, CORNEA, V30, P765, DOI 10.1097/ICO.0b013e318201467c (28)	CORNEA	CORNEA	Ophthalmology
1	Cheung AY, 2020, CORNEA, V39, P980, DOI 10.1097/ICO.0000000000002329 (29)	CORNEA	CORNEA	Ophthalmology
1	Cheung AY, 2017, CURR OPIN OPHTHALMOL, V28, P377, DOI 10.1097/ICU.0000000000000374 (30)	CURR OPIN OPHTHALMOL	CURRENT OPINION IN OPHTHALMOLOGY	Ophthalmology
1	Chong PP, 2017, CLIN THER, V39, P1581, DOI 10.1016/j.clinthera.2017.07.005 (31)	CLIN THER	CLINICAL THERAPEUTICS	Pharmacology & Pharmacy
1	Daya SM, 2000, CORNEA, V19, P443, DOI 10.1097/00003226-200007000-00007 (32)	CORNEA	CORNEA	Ophthalmology
1	Eleiwa T, 2021, J AAPOS, V25, P232, DOI 10.1016/j.jaapos.2021.04.002 (33)	J AAPOS	JOURNAL OF AAPOS	Ophthalmology; Pediatrics
1	Eslani M, 2017, CORNEA, V36, P26, DOI 10.1097/ICO.0000000000000970 (34)	CORNEA	CORNEA	Ophthalmology
1	Phylactou M, 2021, BRIT J OPHTHALMOL, V105, P893, DOI 10.1136/bjophthalmol-2021-319338 (35)	BRIT J OPHTHALMOL	BRITISH JOURNAL OF OPHTHALMOLOGY	Ophthalmology
1	Prendecki M, 2021, ANN RHEUM DIS, V80, P1322, DOI 10.1136/annrheumdis-2021-220626 (36)	ANN RHEUM DIS	ANNALS OF THE RHEUMATIC DISEASES	Rheumatology
1	Sel S, 2012, J IMMUNOL METHODS, V381, P23, DOI 10.1016/j.jim.2012.04.005 (37)	J IMMUNOL METHODS	JOURNAL OF IMMUNOLOGICAL METHODS	Biochemical Research Methods, Immunology
1	Stumpf J, 2021, LANCET REG HEALTH-EU, V9, DOI 10.1016/j.lanepe.2021.100178 (38)	LANCET REG HEALTH-EU	LANCET REGIONAL HEALTH-EUROPE	Health Care Sciences & Services, Public, Environmental & Occupational Health
…	…	…	…	…
82841	Bernard A, 2020, EUR J OPHTHALMOL, V30, P874, DOI 10.1177/1120672119856943 (39)	EUR J OPHTHALMOL	EUROPEAN JOURNAL OF OPHTHALMOLOGY	Ophthalmology
82841	Carvounis PE, 2004, OPHTHALMOLOGY, V111, P1023, DOI 10.1016/j.ophtha.2003.08.032 (40)	OPHTHALMOLOGY	OPHTHALMOLOGY	Ophthalmology
82841	Kaffenberger BH, 2017, J AM ACAD DERMATOL, V76, P140, DOI 10.1016/j.jaad.2016.08.014 (41)	J AM ACAD DERMATOL	JOURNAL OF THE AMERICAN ACADEMY OF DERMATOLOGY	Dermatology
82841	May K, 2021, CASE REP INFECT DIS, V2021, DOI 10.1155/2021/5565900 (42)	CASE REP INFECT DIS	CASE REPORTS IN INFECTIOUS DISEASES	Infectious Diseases
82841	Raja H, 2016, SURV OPHTHALMOL, V61, P726, DOI 10.1016/j.survophthal.2016.03.011 (43)	SURV OPHTHALMOL	SURVEY OF OPHTHALMOLOGY	Ophthalmology
82841	Ramgopal S, 2016, J NEUROL, V263, P500, DOI 10.1007/s00415-015-8007-x (44)	J NEUROL	JOURNAL OF NEUROLOGY	Clinical Neurology
82841	Schwenkenbecher P, 2017, BMC INFECT DIS, V17, DOI 10.1186/s12879-016-2112-z (45)	BMC INFECT DIS	BMC INFECTIOUS DISEASES	Infectious Diseases
82841	Sood SK, 2015, INFECT DIS CLIN N AM, V29, P281, DOI 10.1016/j.idc.2015.02.011 (46)	INFECT DIS CLIN N AM	INFECTIOUS DISEASE CLINICS OF NORTH AMERICA	Immunology, Infectious Diseases
82841	WINTERKORN JMS, 1990, SURV OPHTHALMOL, V35, P191, DOI 10.1016/0039-6257(90)90089-E (47)	SURV OPHTHALMOL	SURVEY OF OPHTHALMOLOGY	Ophthalmology

A statistical analysis was conducted on the disciplinary categories of the obtained references, resulting in the distribution of disciplinary categories of literature citations in the field of ophthalmology (**Table 2**). A total of 250 disciplines from the WOS were included in the analysis, with the top ten disciplines in terms of the number of citations being Ophthalmology, Neurosciences, Surgery, Multidisciplinary Sciences, Biochemistry & Molecular Biology, Psychology, Medicine: Genetics & Heredity, Cell Biology, Genetics & Heredity, Clinical Neurology, and others. Conversely, disciplines with the fewest citations, such as Medieval & Renaissance Studies, Logic, Theater, Religion, and others, are mostly in the humanities and social sciences. This indicates a lesser degree of integration between the field of ophthalmology and these fields.

**Table 2:** Distribution of disciplinary categories of literature citations in ophthalmology (part).

Web of Science Category	Number of entries
Ophthalmology	1943356
Neurosciences	242383
Surgery	189787
Multidisciplinary Sciences	139059
Biochemistry & Molecular Biology	139015
Psychology	99453
Medicine: General & Internal	93873
Cell Biology	93317
Genetics & Heredity	73246
Clinical Neurology	59207
Pharmacology & Pharmacy	54927
Medicine, Research & Experimental	52218
Immunology	46939
Endocrinology & Metabolism	46649
Pediatrics	45998
Physiology	37207
Oncology	36479
Public, Environmental & Occupational Health	30539
Optics	28523
Radiology, Nuclear Medicine & Medical Imaging	28091
Psychology, Experimental	24907
Biology	24075
Biochemical Research Methods	23513
Biotechnology & Applied Microbiology	19179
Pathology	18666
Hematology	18278
Dermatology	17533
Peripheral Vascular Diseases	17238
Biophysics	17225
Behavioral Sciences	17087
…	…
Poetry	5
Engineering, Petroleum	4
Literary Theory & Criticism	4
Religion	4
Literature, German, Dutch, Scandinavian	2
Literature, Romance	2
Literature, Slavic	2
Theater	2
Logic	1
Medieval & Renaissance Studies	1

### 3.2 Interdisciplinary literature findings in ophthalmology based on the Rao-Stirling index

The mapping results of the “literature reference- discipline category” employed in the construction of the co-occurrence matrix of “literature- literature reference discipline category”. The inter-discipline citation and cited similarity matrix was used as a reference for calculating the inter-discipline similarity distance. A matrix of disciplinary distributions for literature citations in the field of ophthalmology was constructed using VBA. The Web of Science Categories similarity matrix developed by Chavam D et al. was employed in R programming to compute the variety, balance, disparity, and Rao-Stirling diversity index for each literature item in the field of ophthalmology (48). **Table 3** presents the results of the literature in the field of ophthalmology arranged in descending order based on the Rao-Stirling index.

**Table 3:** Results of interdisciplinary measurement of literature in the field of ophthalmology (part).

ID	Title	Rao-Stirling	Variety	Balance	Disparity
25073	Airborne pathogen projection during ophthalmic examination	0.91	30	0.97	0.96
78957	Highly cited publication performance in the ophthalmology category in the Web of Science database: a bibliometric analysis	0.91	34	0.92	0.96
40581	Edge detection and mathematic fitting for corneal surface with Matlab software	0.91	26	0.96	0.95
21660	Characterization of human retinal vessel arborisation in normal and amblyopic eyes using multifractal analysis	0.91	24	0.96	0.96
332	Effects of Immersive Virtual Reality Headset Viewing on Young Children: Visuomotor Function, Postural Stability, and Motion Sickness	0.90	26	0.92	0.97
17444	Improving the pilot selection process by using eye-tracking tools	0.90	15	0.99	0.97
20872	Deep Learning-Assisted Multiphoton Microscopy to Reduce Light Exposure and Expedite Imaging in Tissues With High and Low Light Sensitivity	0.90	37	0.93	0.95
79917	Mobile eye tracking applied as a tool for customer experience research in a crowded train station	0.90	26	0.91	0.96
78904	Grading the Severity of Damage to the Perijunctional Actomyosin Ring and Zonula Occludens-1 of the Corneal Endothelium by Ensemble Learning Methods	0.90	37	0.93	0.95
76638	Automatic Identification and Segmentation of Orbital Blowout Fractures Based on Artificial Intelligence	0.90	18	0.96	0.96
…	…	…	…	…	…
82824	Workforce separation among ophthalmologists before and during the COVID-19 pandemic	0	0	0	0

**Figure 2:**
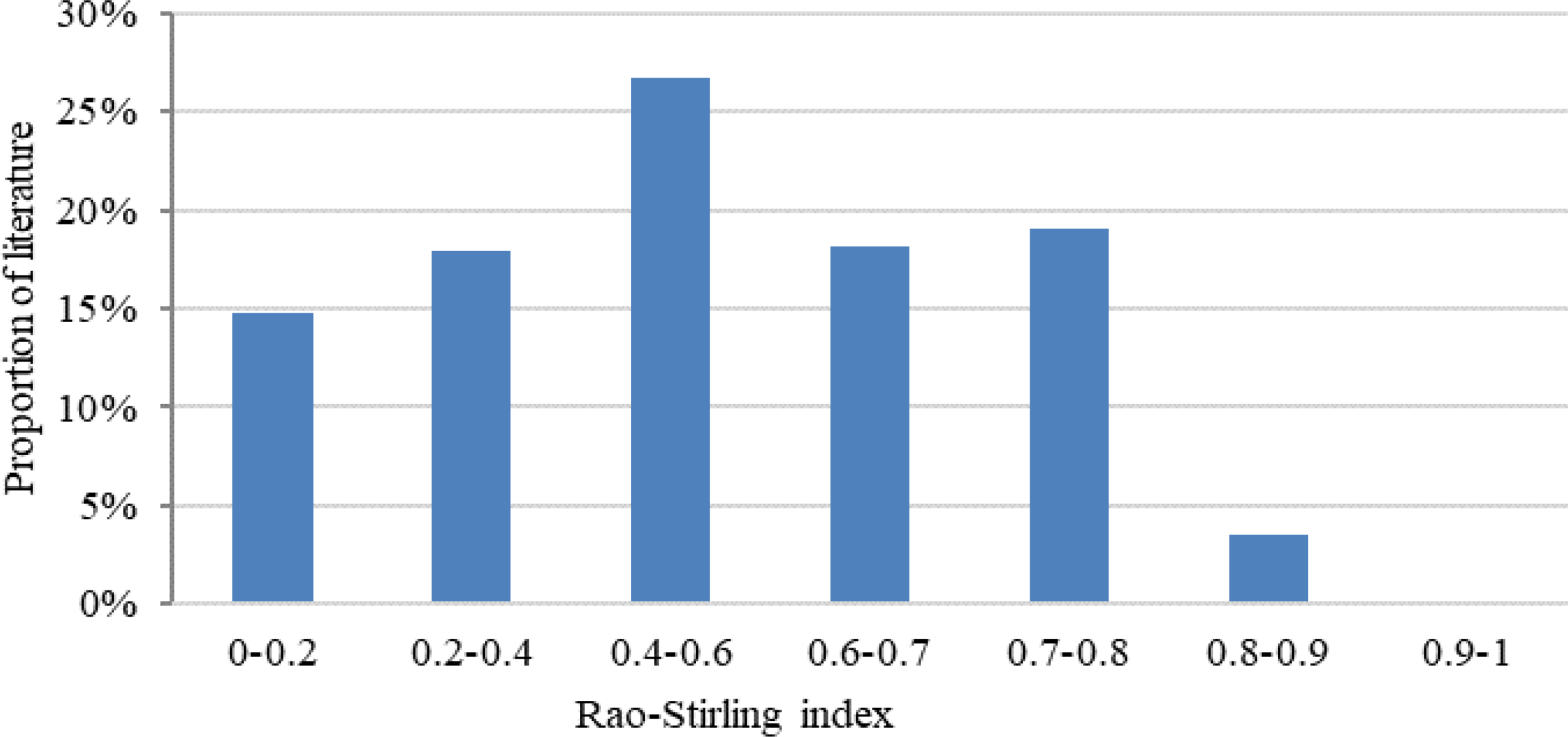
Frequency histogram of Rao-Stirling index in the literature on ophthalmology.

A frequency analysis was conducted on the distribution of the Rao-Stirling index in the literature of ophthalmology, resulting in the generation of a histogram illustrating the frequency distribution (**Figure 2**). An analysis of the frequency distribution histogram and relevant literature revealed that most publications in ophthalmology have Rao-Stirling index values primarily between 0.2 and 0.7. Taking into account the distribution of Rao-Stirling index values and the need to balance the inclusion of a sufficient number of interdisciplinary articles with a significant level of interdisciplinary engagement (49), a threshold of 0.7 was chosen. As a result, 18,573 articles with a Rao-Stirling index above 0.7 were selected for further investigation into interdisciplinary topics within ophthalmology.

### 3.3 Subject identification of interdisciplinary articles in ophthalmology based on LDA model

**Figure 3:**
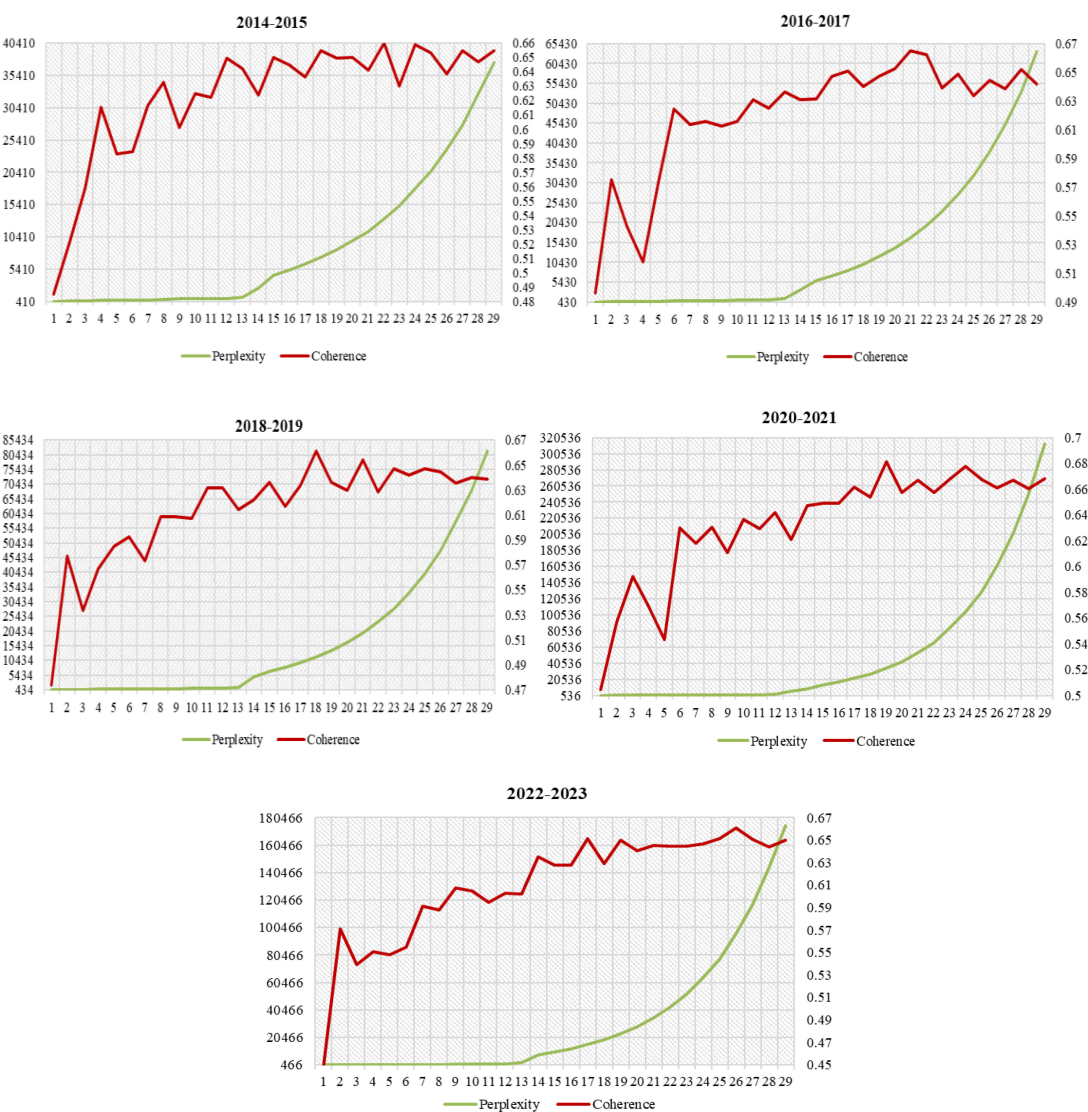
Topic coherence and topic perplexity under different time windows.

We extract the author keywords, keywords plus of the screened disciplinary crossover literature in the field of ophthalmology, using the LDA model to conduct a subject identification study of the interdisciplinary articles in the field of ophthalmology. This research employed the LDA model technique utilizing Gensim version 4.3.1. The core parameters were established as follows: a grid search was performed to determine the optimal number of topics, ranging from 2 to 30, with validation achieved through a joint assessment of perplexity and coherence scores. The model underwent training over 20 passes of the corpus, with each pass comprising 50 default iterations. The convergence criterion was set to a change in log-likelihood between successive iterations of less than 1e-6. A symmetric Dirichlet prior (α=50/k) and a fixed word prior (η=0.1) were utilized, and consistency in initialization was maintained by setting the random_state to 1. This configuration of parameters effectively balances computational efficiency with model interpretability, aligning with the generative framework of LDA while ensuring reproducibility through the explicit specification of hyperparameters and the use of fixed random seeds. The topic coherence and topic perplexity curves are shown in **Figure 3**. The points where the topic consistency rises and the perplexity decreases was selected as the reference point for determining the number of topics (50). A comparison analysis was conducted to determine the final number of topics under the time window. From the topic coherence and topic perplexity curves of each time window, it can be observed that the number of themes present in each era is as follows: 12 themes in 2014-2015, 11 themes in 2016-2017, 11 themes in 2018-2019, 10 themes in 2020-2021, and 9 themes in 2022-2023.

In conjunction with the quantity of topics identified within each temporal interval and the associated keywords, a process of manual annotation is conducted. To improve the validity and robustness of the LDA topic labels, a team of annotators with expertise in ophthalmology was recruited for the labeling process. Each annotator independently assigned labels to the topics, and the consistency of their labeling was assessed using Cohen’s Kappa statistic. The Kappa values obtained, which ranged from 0.78 to 0.91, indicated substantial to nearly perfect agreement among the annotators, thereby confirming the reliability of the topic labels. The results are presented in **Table 4**, with additional details available in **Appendix A**.

**Table 4:** Distribution of topic-high probability feature words under each time window.

Time window	Final number of topics	Tags coding	Topic Tags	Cohen’s Kappa
2014-2015	12	1-1	Neuroprotective effect and mechanism of vitamin D in various retinal and optic neuropathies	0.85
		1-2	Study on the changes of retinal blood flow and its effect on visual function under different visual impairment models	0.90
		1-3	Mechanisms of angiogenesis and inflammation regulation and efficacy of treatment in age-related macular degeneration and related eye diseases	0.83
		1-4	The mechanism of apoptosis induced by TNF-α in ocular diseases	0.81
		1-5	Application of antioxidant in relieving oxidative stress and protecting retinal function	0.87
		1-6	An epidemiological and mechanism study of the effects of retinal diseases	0.91
		1-7	The mechanism of photoreceptor function, inflammatory factors and corneal neovascularization in diabetes-related keratopathy	0.83
		1-8	Multidimensional study of eye health and Visual function: Genetic and Environmental Factors from corneal regeneration to visual cognition	0.85
		1-9	Study on the pathogenesis, visual function and therapeutic potential in retinopathy of prematurity	0.84
		1-10	Study on the effects of diabetic retinopathy on visual attention and motion perception and its neural mechanism and intervention strategies	0.83
		1-11	Comprehensive study on the influence of eye disease on visual function and exploration of potential treatment strategies	0.82
		1-12	Multi-dimensional gene therapy and cell therapy for eye diseases	0.79
2016-2017	11	2-1	Mechanism of retinal ganglion cell injury and repair in ocular diseases and the strategy of visual function restoration	0.88
		2-2	Research on corneal health and protection of visual function in the context of aging and neurodegeneration	0.82
		2-3	Innovative research in multimodal therapy and individualized strategies for ophthalmic disease management	0.85
		2-4	Visual function restoration and assistive technology in patients with low vision: a comprehensive evaluation based on eye movement, image analysis and retinal imaging	0.83
		2-5	Multi-dimensional study of visual system lesions and molecular mechanisms	0.86
		2-6	Study on cell biology and biochemistry of ocular diseases	0.84
		2-7	Study on mechanism of retinopathy and vision protection strategy	0.85
		2-8	Angiogenesis and inflammatory regulation in diabetes retinopathy and epigenetic study of dexamethasone intervention	0.78
		2-9	Study on the mechanism and influence of VEGF in diabetes retinopathy and complications	0.89
		2-10	Study on the mechanism of retinal nerve protection and intervention for visual impairment	0.91
		2-11	Multi-dimensional study of ocular cytology	0.82
2018-2019	11	3-1	Mechanism and intervention strategy of oxidative stress and visual impairment in ocular diseases	0.80
		3-2	Study on the regulatory mechanisms of inflammation, neovascularization and apoptosis in ocular diseases and their effects on visual function	0.80
		3-3	Multimodal evaluation of ocular structure and function and its application and intervention in common ocular diseases	0.84
		3-4	Oxidative stress and gene regulatory networks in retinal degenerative diseases	0.86
		3-5	Studies of myopia and visual impairment: from molecular mechanisms to visual function assessment and intervention strategies	0.88
		3-6	Multimodal imaging and molecular mechanism of eye diseases and its application in vision protection and diagnosis	0.89
		3-7	Study of the global epidemiology, prevention and treatment strategies of eye diseases	0.83
		3-8	Mechanisms and neuroprotective strategies of retinal cells and their associated signaling pathways in visual perception and ocular diseases	0.85
		3-9	Multi-dimensional study of retinal diseases: from neuroprotective mechanisms to diagnostic markers and therapeutic targets	0.87
		3-10	Study on pathological mechanism and innovative treatment strategy of corneal diseases and diabetic retinopathy	0.82
		3-11	Study of the pathogenesis, diagnosis, treatment of ocular diseases	0.87
2020-2021	10	4-1	Study on the pathogenesis, diagnosis and intervention strategies of diabetic retinopathy and its complications	0.83
		4-2	Comprehensive study on the relationship between innate immunity and visual system development and function	0.80
		4-3	Pathogenesis analysis, diagnostic screening, and development of personalized treatment strategies for eye diseases based on machine learning	0.81
		4-4	Comprehensive study of the effects of Graves’ disease and other eye diseases on visual adaptation, holistic perception, and mental health in children	0.81
		4-5	Study on the mechanism of exosomes in retinal inflammation, oxidative stress and treatment in uveitis and related ocular diseases	0.83
		4-6	The regulatory mechanism of autophagy and cell proliferation, plasticity and angiogenesis in anterior segment disease	0.82
		4-7	Mechanisms of interaction of ocular diseases with pathogen infections and environmental factors	0.79
		4-8	Meta-analysis of ophthalmic diagnostic techniques on retinopathy	0.82
		4-9	Cytological and epidemiology study in the treatment of eye diseases	0.80
		4-10	Mechanisms of retinal ganglion cell protection and optic nerve regeneration driven by virtual reality and eye tracking technology	0.78
2022-2023	9	5-1	Multi-dimensional study on the pathological mechanism and visual perception of ocular diseases based on optical coherence tomography	0.83
		5-2	Epidemiological studies and public health responses to the impact of COVID-19 on eye health	0.82
		5-3	Multi-dimensional study of ophthalmic diseases: Innovative exploration from pathological mechanism to clinical diagnosis and treatment	0.85
		5-4	Comprehensive study on the pathological mechanism, visual function and drug intervention of glaucoma	0.83
		5-5	Comprehensive study of diabetic retinopathy, visual function and related risk factors	0.82
		5-6	Comprehensive study of eye disease diagnosis and vision protection based on artificial intelligence	0.81
		5-7	Multi-dimensional study of eye disease: pathological mechanisms and intervention strategies	0.84
		5-8	Multi-omics study of COVID-19-associated retinal microenvironmental alterations and inflammatory mechanisms	0.87
		5-9	Study on pathogenesis, biomarkers and treatment strategies of diabetic retinopathy and related eye diseases	0.86

**Figure 4:**
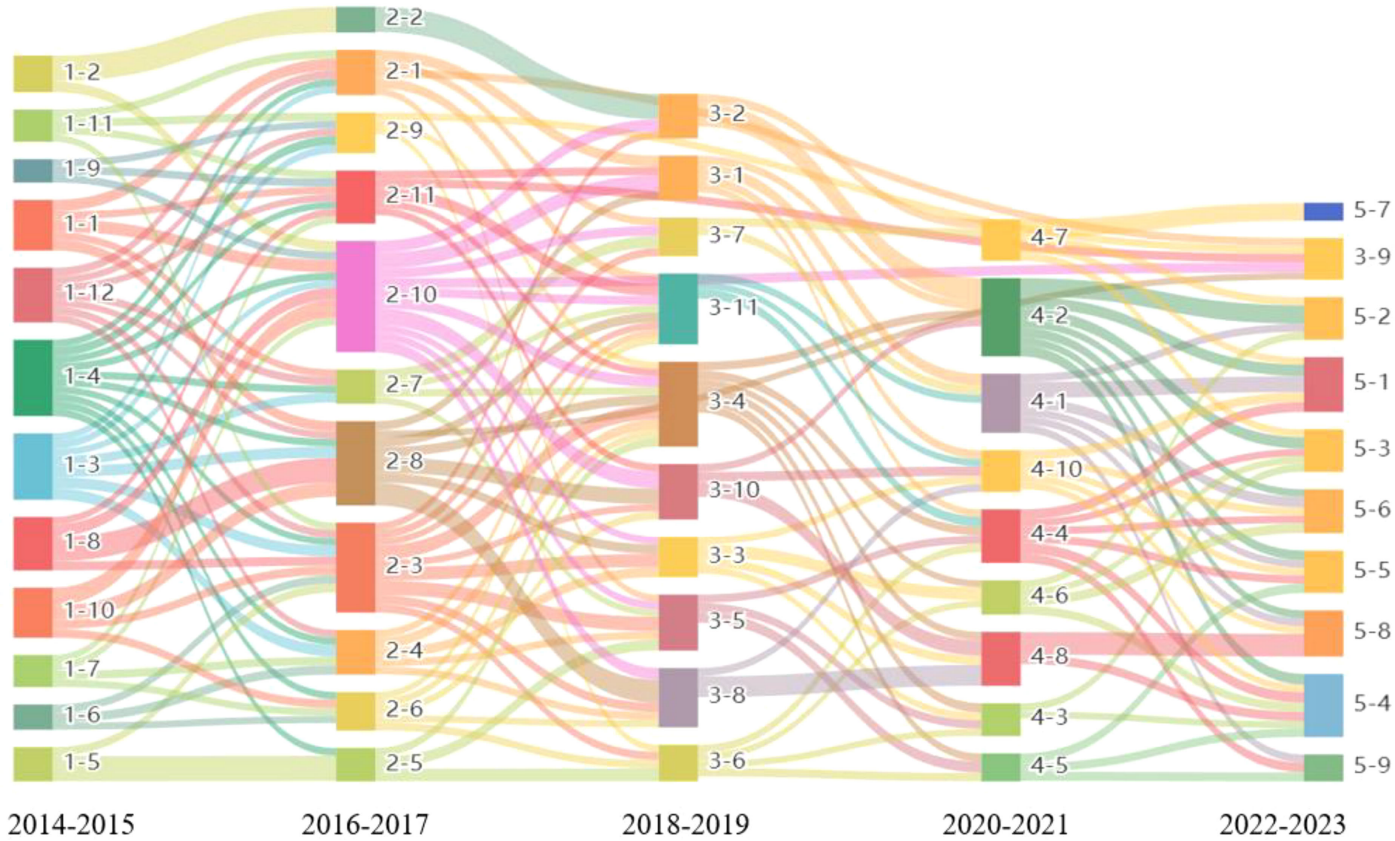
Theme evolution Sankey diagram.

Combined with the label coding columns in **Table 4**, The evolutionary relationships among topics are established by computing the cosine distance values between the topics and the associated terms identified within consecutive time intervals. The mean similarity value of all topics within a designated time window is utilized as a threshold (51–54). When the similarity value between topics exceeds the predefined threshold value of 0.212, an evolutionary relationship among the topics can be inferred. Ultimately, the evolutionary trajectory is delineated by integrating the outcomes of the cosine value computations. The results are shown in **Figure 4**. The horizontal axis of the graph delineates a chronological progression, extending from left to right, and is segmented into five distinct periods (2014-2015, 2016-2017, 2018-2019, 2020-2021, 2022-2023). The vertical axis features various colored rectangles that signify different research topics, each assigned a unique identifier (e.g., 1-1, 1-2, etc.). The dimensions of the element blocks correspond to the relative prominence of each topic during the specified period, with larger blocks indicating greater significance. Additionally, connecting lines are employed to illustrate the thematic evolution across different stages, with the thickness of these lines representing the degree of transfer or correlation between themes. A greater thickness of the lines denotes a stronger degree of transfer or correlation.

The evolutionary path is “1-1, 1-2, 1-3, 1-5, 1-6, 1-9,1-10→2-1, 2-4, 2-7, 2-8, 2-9, 2-10→3-4, 3-8, 3-10→4-1, 4-5, 4-8, 4-10→5-5, 5-8, 5-9”. This trajectory illustrates the evolution of research on retinal diseases from a focus on singular mechanisms to a more integrated, multidimensional approach. In 2014-2015, researchers primarily concentrated on elucidating the fundamental pathological mechanisms and contributing factors associated with retinal diseases. They established a framework for immunomodulatory interventions in conditions such as retinitis pigmentosa (55, 56) and age-related macular degeneration (57–59). In 2016-2017, the emphasis of research began to transition towards optic nerve repair and the regulation of epigenetic factors, leading to significant advancements in understanding the mechanisms underlying retinal ganglion cell regeneration (60, 61) and the identification of precise corticosteroid dosing targets (62, 63). The period of 2018-2019 witnessed the widespread adoption of systems biology methodologies in retinal disease research, enabling scholars to delineate critical pathways involved in antioxidant defense through the construction of gene regulatory networks (64, 65). This period also saw the proposal of innovative combinations of therapeutic targets, thereby offering new avenues for the treatment of retinal disorders (66, 67). The years 2020-2021 marked a pivotal moment, as the rapid advancement of information technology facilitated the integration of artificial intelligence (68, 69) and virtual reality into the precision diagnosis and mechanistic study of retinal diseases (70, 71). This integration is anticipated to yield significant improvements in therapeutic strategy optimization, diagnostic model development, and the assessment of neural regeneration. In 2022-2023, the repercussions of the COVID-19 outbreak, coupled with advancements in multi-omics technology, prompted researchers to investigate the molecular mechanisms underlying COVID-19 retinopathy (72, 73) and to establish an early warning model for diabetic retinopathy (74, 75). This progress is expected to enhance individualized diagnostic and therapeutic approaches, bridging the gap between theoretical research and clinical application. Overall, the evolution of retinal disease research has been characterized by substantial shifts in research paradigms, technological advancements, and clinical applications, progressively moving towards the analysis of multi-signal interactions, the in-depth application of cutting-edge technologies, and the establishment of a precision medicine framework.

The evolutionary path is “1-1, 1-2, 1-3, 1-5, 1-6, 1-9,1-10→2-1, 2-4, 2-7, 2-8, 2-9, 2-10→3-4, 3-8, 3-10→4-1, 4-5, 4-8, 4-10→5-5, 5-8, 5-9”. Expanding on the concept of ophthalmic disease, research on multidimensional studies of the entire visual system, multimodal treatments, and multimodal evaluations is developing more rapidly. In 2014-2015, research efforts concentrated on elucidating the molecular mechanisms underlying TNF-induced apoptosis (76, 77), this discovery has identified potential targets for anti-inflammatory therapies. During this period, advancements in gene and cell therapy technologies emerged, alongside significant breakthroughs in the clinical application of anti-VEGF (vascular endothelial growth factor) drugs (78, 79) and stem cell transplantation (80, 81), marking the onset of precision ophthalmic treatment. In 2016-2017, the focus of research transitioned towards the deepening of technological applications and the development of individualized treatment strategies. Multi-modal treatment plans, tailored to disease staging and patient characteristics, markedly enhanced therapeutic efficacy. In-depth analyses of the role of VEGF in fundus neovascularization expedited the application of anti-VEGF therapies (82, 83). Additionally, the elucidation of the interactions between microglia and retinal ganglion cells, as well as the regulation of metabolic processes, introduced novel perspectives for neuroprotective treatments (84, 85). From 2018 to 2019, advancements in multimodal imaging technologies significantly improved the diagnostic accuracy of ocular tumors and vascular lesions, thereby facilitating the development of etiological diagnostic methods (86, 87). Global epidemiological studies clarified the burden of conditions such as myopia (88, 89) and dry eye (90, 91), providing a foundation for public health policy formulation. In 2020-2021, research began to explore the roles of innate immunity and artificial intelligence as emerging focal points. Investigations into the mechanisms by which innate immune cells contribute to eye diseases have the potential to identify targets for immunomodulatory therapies (92, 93). Concurrently, machine learning algorithms are being developed to facilitate early screening and personalized treatment recommendations for eye diseases (94, 95). The intersection of cytology and epidemiology underscores the synergistic effects of pathogenic bacterial infections and environmental factors, thereby informing public health strategy development (96, 97). By 2022 to 2023, the impact of the COVID-19 pandemic on ocular health gained prominence, researchers advocate for the integration of ophthalmology within infectious disease prevention and control frameworks (98, 99), and analyze the adverse effects of vaccination on the eye (100, 101). The application of artificial intelligence technologies to synthesize multimodal data aims to optimize diagnostic and therapeutic decision-making of eye diseases (102, 103). Furthermore, multidimensional intervention strategies that combine gene editing (104, 105), cellular therapy (106, 107), and drug discovery (108, 109) present new avenues for addressing refractory eye diseases. This progression illustrates a significant evolution in ophthalmic research, transitioning from basic mechanistic analyses to clinical technological innovations, from singular treatment mechanisms to multi-faceted interventions, and from localized studies to global collaborations, thereby establishing a robust foundation for the prevention and treatment of ocular diseases.

## 4 Discussion

This paper provides an in-depth identification and evolutionary analysis of the cross-disciplinary themes in the field of Ophthalmology based on the Rao-Stirling index and the LDA model. From the distribution of cross-disciplinary fields in the discipline of Ophthalmology, the research in the discipline of Ophthalmology shows a significant multidisciplinary cross-development trend. From the point of view of the evolution of cross-disciplinary themes in Ophthalmology, with the deepening of medical research and the continuous progress of science and technology, the research themes in the field of Ophthalmology have experienced significant evolution and development. Based on this, this paper puts forward the following suggestions to promote the development of cross-thematic integration in the discipline of Ophthalmology.

### 4.1 Advancements in ophthalmology through interdisciplinary collaboration

The future of ophthalmology is increasingly leaning towards interdisciplinary collaboration. The complexity of modern eye diseases, such as age-related macular degeneration and diabetic retinopathy, necessitates moving beyond the confines of a single discipline. This calls for the development of a comprehensive research and practice framework that integrates multiple dimensions. This adaptive approach indicates that future technological advancements will rely not only on the development of ophthalmic devices and diagnostic and therapeutic technologies but also on creating a robust interdisciplinary innovation ecosystem. This ecosystem aims to dismantle disciplinary silos by establishing structured knowledge-sharing mechanisms, such as cross-disciplinary academic communities and joint laboratory platforms, as well as resource-sharing networks that integrate clinical data, technological tools, and translational pathways. This integration will foster collaboration between ophthalmology and fields like Neuroscience, Surgery, Biochemistry, Psychology, Cell Biology, and Genetics. Through this innovative network, ophthalmology is poised to undergo significant transformations in precision and regenerative medicine, ultimately leading to a collaborative innovation system that encompasses the entire spectrum of “Prevention - Diagnosis - Treatment - Rehabilitation.”

### 4.2 Advancements in ophthalmology through innovation in scientific research funding projects

In response to the trend of interdisciplinary integration within the field of ophthalmology, it is essential to develop a systematic strategy for funding research projects. First, the establishment of specialized interdisciplinary funds is necessary, with a focus on supporting collaborative research between ophthalmology and fields such as neuroscience, biomedical engineering, and artificial intelligence. This approach aims to effectively guide scholars from related disciplines in forming interdisciplinary teams, thereby overcoming the limitations associated with single-discipline research. Second, a multi-party joint funding mechanism should be established, creating a collaborative model involving funding agencies, enterprises, and medical institutions. This initiative encourages the formation of joint funding communities between government funds and medical device companies, as well as hospitals. For instance, in the development of precision diagnostic equipment, companies can provide engineering expertise and preliminary research funding, while hospitals contribute clinical data and validate application scenarios. Funding agencies can establish “clinical translation-oriented” special funds to facilitate the acceleration of the entire chain from “technology validation to product pilot testing to clinical registration.” This approach addresses the resource limitations of individual entities and shortens the cycle of research translation. Third, it is crucial to enhance the interdisciplinary evaluation system by developing a professional review team through a combination of “training, recruitment, and dynamic assessment.” This strategy aims to mitigate biases associated with single disciplines, ensuring that groundbreaking projects receive fair evaluations and dismantling the barriers that inhibit innovation across disciplines.

### 4.3 Reform of ophthalmology education within a multidimensional research framework

The profound transformation of the paradigm in ophthalmic research is driving a transition in the medical education system towards a more integrated and multidimensional approach. Traditional modular courses, which focus on individual diseases, have become inadequate in meeting the contemporary demands for interdisciplinary research talent in ophthalmology due to rigid disciplinary barriers and a disconnect between theory and practice. In this context, the establishment of an integrated educational ecosystem centered on the systematic analysis of disease mechanisms emerges as a necessary solution. This ecosystem facilitates the transition of ophthalmic education from fragmented knowledge dissemination to comprehensive skill development through the deep coupling of interdisciplinary knowledge frameworks and a capability matrix encompassing “basic research - technological innovation - clinical translation.”

In the restructuring of the curriculum, it is essential to enhance the integration of knowledge across various dimensions. For instance, in the design of the teaching framework for diabetic retinopathy, the curriculum can be divided into modules focusing on metabolic-vascular pathological mechanisms, immune-neurological pathology, and technical applications with precision diagnostics. This structure enables students to acquire comprehensive skills in “metabolic control - imaging assessment - minimally invasive treatment,” thereby constructing an interdisciplinary cognitive framework that connects “metabolic abnormalities - vascular damage - neuroimmune interactions” and facilitates a progression in capabilities from phenotype recognition to mechanistic analysis. Additionally, in light of the significant global disease burden posed by diabetic retinopathy, the educational framework could incorporate a module on “Ophthalmic Epidemiology and Global Health,” which would encompass multidimensional etiological research, the formulation of stratified intervention strategies, and the development of global governance competencies. This deeply integrated teaching system, which combines medical, engineering, and theoretical knowledge, will effectively transcend disciplinary boundaries and provide essential support for the advancement of ophthalmology.

### 4.4 Editorial adaptations for interdisciplinary scholarship

The rise of interdisciplinary research within the domain of ophthalmology highlights the imperative for academic journals to revise their editorial policies. To effectively navigate this challenge, journals should consider implementing several critical modifications. Firstly, they ought to broaden their scope to encompass interdisciplinary research that merges molecular biology with data science or engineering, exemplified by exosome-based therapies in conjunction with AI diagnostics. This approach would attract pioneering research and advance the field of ophthalmology. Secondly, journals should adopt more flexible peer review processes, recruit reviewers from a variety of disciplines, or implement multi-stage reviews to ensure a comprehensive and equitable assessment of the quality of interdisciplinary research. Finally, the creation of dedicated sections or special issues for the publication of research that transcends conventional boundaries would significantly enhance the visibility of interdisciplinary studies. The rapid integration of COVID-19-related ophthalmic research into high-impact journals serves as a pertinent example of how journals can adapt to emerging interdisciplinary topics, thereby promoting clinical and scientific progress. By expanding their scope, embracing flexible review processes, and establishing dedicated publication avenues, journals not only reflect the interdisciplinary trend but also play a proactive role in steering the evolution of ophthalmological research towards greater integration and innovation.

## 5 Conclusions

In comparison to previous interdisciplinary bibliometric studies within the fields of medicine and ophthalmology, the present research offers several notable contributions. Firstly, it employs the Rao-Stirling index, which uniquely incorporates considerations of diversity, equilibrium, and differentiation across various disciplines, to assess the extent of interdisciplinary engagement within the ophthalmology literature. Secondly, by combining LDA model with the Rao-Stirling index, this study not only provides a quantitative assessment of interdisciplinary collaboration but also facilitates a comprehensive analysis of the evolving trajectories of interdisciplinary themes over time. This multifaceted methodology yields new insights into the dynamics of interdisciplinary research in ophthalmology.

Nevertheless, this study is not without its limitations, and there are challenges that must be addressed in future research. Primarily, this investigation focuses on publication metrics derived from the Web of Science, thereby omitting significant non-traditional contributions such as software tools, clinical guidelines, and translational research. Future research endeavors will aim to provide a more holistic overview of interdisciplinary collaboration in ophthalmology by incorporating a wider array of contributions. Additionally, to evaluate the effectiveness of interdisciplinary collaborations—particularly in terms of positive patient outcomes—qualitative case studies, surveys, or interviews will be necessary to provide more robust evidence. Furthermore, future studies should explore the integration of comprehensive methodologies that combine the Rao-Stirling index, LDA model, and qualitative approaches. Lastly, as advancements in artificial intelligence and precision medicine continue to develop, our findings have effectively identified these emerging research hotspots. However, the study does not address the practical challenges associated with the integration of these technologies into routine clinical practice. Therefore, future research should focus on exploring implementation strategies, comparative efficacy, and the policy implications of incorporating emerging technologies such as AI and precision medicine into standard ophthalmological workflows.

## Data Availability

The original contributions presented in the study are included in the article/Supplementary Material. Further inquiries can be directed to the email luchunji0108@163.com.
