# Supplementary material for: Research on interdisciplinary measurement and evolutionary path in the field of ophthalmology
Source: Front Ophthalmol (Lausanne). 2025 Jun 6;5:1578956. doi: 10.3389/fopht.2025.1578956 (PMC12186160; doi:10.3389/fopht.2025.1578956)
Supplement: Supplementary file 1 [file SupplementaryFile1.docx]

**Appendix A**

*Distribution of topic-high probability feature words under each time window*

| **Time window** | **Tags coding** | **Topic Tags** | **Top 30 high-probability feature words for the topic(part)** |
| --- | --- | --- | --- |
| 2014-2015 | 1-1 | Neuroprotective effect and mechanism of vitamin D in various retinal and optic neuropathies | neuroprotection, retinitis pigmentosa, material perception, optic neuropathy, diabetes, vitamin D, retinal degeneration, macula, uveal melanoma, glaucoma, corneal confocal microscopy, diabetic retinopathy, diabetic neuropathy, GFAP |
|  | 1-2 | Study on the changes of retinal blood flow and its effect on visual function under different visual impairment models | retinal, blood flow, natural images, diabetic retinopathy, color vision, mice, glaucoma, dry eye syndrome, zebrafish, object recognition, erythropoietin, cornea, neovascularization, blood-retinal barrier, hyperoxia |
|  | 1-3 | Mechanisms of angiogenesis and inflammation regulation and efficacy of treatment in age-related macular degeneration and related eye diseases | age-related macular degeneration, choroidal neovascularization, vascular endothelial growth factor, endophthalmitis, microglia, uveitis, retina, optical coherence tomography, proliferative diabetic retinopathy, central serous chorioretinopathy, retinoblastoma, cataract surgery, orbit, choroid, autoimmunity |
|  | 1-4 | The mechanism of apoptosis induced by TNF-α in ocular diseases | TNF-alpha, apoptosis, keratoconus, allergic conjunctivitis, age-related macular degeneration, contextual modulation, retinal pigment epithelial cells, macular pigment, mitochondria, protein kinase C, keratocytes, retinal vessels, proliferative vitreoretinopathy, afterimages, neutrophils, ERstress |
|  | 1-5 | Application of antioxidant in relieving oxidative stress and protecting retinal function | oxidative stress, retina, glaucoma, age-related macular degeneration, retinal pigment epithelium, AMD, apoptosis, optical coherence tomography antioxidant, adaptive optics, retinal ganglion cell, pharmacokinetics, peripheral vision, complement, perception, ganglion cells |
|  | 1-6 | An epidemiological and mechanism study of the effects of retinal diseases | epidemiology, retina, visual search, eye movements, low vision, driving, genetics, visual field, cataract, keratitis, OCT, proteomics, face perception, macular degeneration, mass spectrometry, animal models |
|  | 1-7 | The mechanism of photoreceptor function, inflammatory factors and corneal neovascularization in diabetes-related keratopathy | photoreceptors, corneal neovascularization, aqueous humor, color constancy, cytokines, inflammation, color appearance, meta-analysis, wound healing, human corneal endothelial cells, diabetic retinopathy, brightness, glial fibrillary acidic protein, corneal epithelial cells, nestin |
|  | 1-8 | Multidimensional study of eye health and Visual function: Genetic and Environmental Factors from corneal regeneration to visual cognition | eye, ischemia, corneal wound healing, corneal epithelium, retinal neovascularization, single nucleotide polymorphism, graves' orbitopathy, resveratrol, visual working memory, trachoma, alzheimer's disease, visual cortex, meibomian gland, choroidal thickness, binocular rivalry |
|  | 1-9 | Study on the pathogenesis, visual function and therapeutic potential in retinopathy of prematurity | retinopathy of prematurity, VEGF, macular edema, optic nerve, bevacizumab, visual cycle, light damage, melanopsin, tight junctions, cones, color categories, blood retinal barrier, depression, retinal arterioles, pupil light reflex, prematurity |
|  | 1-10 | Study on the effects of diabetic retinopathy on visual attention and motion perception and its neural mechanism and intervention strategies | diabetic retinopathy, visual attention, retinal nerve fiber layer, lens, optical coherence tomography, cell culture, reactive oxygen species, psychophysics, eye movements, mouse, eye tracking, time perception, aldose reductase, extraocular muscle, ranibizumab, phagocytosis, motion perception |
|  | 1-11 | Comprehensive study on the influence of eye disease on visual function and exploration of potential treatment strategies | RPE, primary open-angle glaucoma, central retinal vein occlusion, electrophysiology, depth perception, trabeculectomy, intraocular pressure, corneal endothelial cell, retinal prosthesis, retinitis pigmentosa, stereopsis, contrast sensitivity function, perceptual learning, SIRT1, diabetic retinopathy |
|  | 1-12 | Multi-dimensional gene therapy and cell therapy for eye diseases | gene therapy, oxygen-induced retinopathy, corneal transplantation, ocular surface inflammation, muller cells, retinal ganglion cells, dry eye, fibrosis, motion perception, retinal degeneration, cell transplantation, graves' ophthalmopathy, monocytes, microarray, ocular trauma |
| 2016-2017 | 2-1 | Mechanism of retinal ganglion cell injury and repair in ocular diseases and the strategy of visual function restoration | retinal ganglion cell, keratitis, color constancy, retinal nerve fiber layer, perceptual learning, graves' orbitopathy, retinal ischemia, glutathione, vision, cell death, microarray, glaucoma, diabetic retinopathy, tear fluid, reactive oxygen species, keratoprosthesis |
|  | 2-2 | Research on corneal health and protection of visual function in the context of aging and neurodegeneration | aging, neurodegeneration, cornea, optic flow, resveratrol, signal detection theory, prevalence, eye drops, trachoma, retina, object recognition, global trachoma mapping project, shape perception, inflammation, oxidative stress, regenerative medicine, superoxide dismutase, gliosis |
|  | 2-3 | Innovative research in multimodal therapy and individualized strategies for ophthalmic disease management | optical coherence tomography, visual cortex, glaucoma, gene therapy, intravitreal injection, vitamin D, histopathology, trabeculectomy, silicone hydrogel, cataract, drug delivery, human tenon's fibroblasts, retinal pigment epithelium, individual differences, open-angle glaucoma, corneal donation, face recognition |
|  | 2-4 | Visual function restoration and assistive technology in patients with low vision: a comprehensive evaluation based on eye movement, image analysis and retinal imaging | low vision, adaptive optics, age-related macular degeneration, saccades, visual search, eye movements, eye tracking, ocular surface, contrast sensitivity, visual field, retinal imaging, electroretinography, face perception, inflammation, retina, retinitis pigmentosa, image analysis, optical coherence tomography |
|  | 2-5 | Multi-dimensional study of visual system lesions and molecular mechanisms | cataract, oxidative stress, color vision, myopia, visual acuity, texture, vitreous humor, age-related macular degeneration, concussion, choroidal neovascularization, uveal melanoma, vergence, mass spectrometry, Rho-kinase, peptide, angiography, binocular vision |
|  | 2-6 | Study on cell biology and biochemistry of ocular diseases | retinopathy, apoptosis, conjunctiva, antioxidant, oxygen-induced retinopathy, trachoma, retinal neovascularization, biofilm, diabetic retinopathy, contact lens, intraocular pressure, normal tension glaucoma, sphingosine-1-phosphate, glaucoma, retina angiogenesis, biomechanics, limbal stem cell deficiency, chlamydia trachomatis |
|  | 2-7 | Study on mechanism of retinopathy and vision protection strategy | retina, optic neuropathy, mitochondria, oxidative stress, material perception, genetics, choroid, ganglion cell complex, diabetic retinopathy, continuous flash suppression, refractive error, inflammation, bruch's membrane, rods, pseudo exfoliation syndrome, glaucoma, visual awareness, toxicity, optical coherence tomography, spatial vision |
|  | 2-8 | Angiogenesis and inflammatory regulation in diabetes retinopathy and epigenetic study of dexamethasone intervention | diabetic retinopathy, retinal degeneration, photoreceptor, neovascularization, inflammation, microglia, dexamethasone, DNA methylation, ischemia, macrophages, autophagy, retina, biomarker, age-related macular degeneration, oxidative stress, retinal pigment epithelium, epigenetics, neuroprotection |
|  | 2-9 | Study on the mechanism and influence of VEGF in diabetes retinopathy and complications | diabetes mellitus, proliferative diabetic retinopathy, diabetic retinopathy, vascular endothelial growth factor, retinopathy, VEGF, treatment, development, retina, inflammation, trabecular meshwork, ganglion cells, visual working memory, vitreous, intraocular pressure, DNA damage, angiogenesis |
|  | 2-10 | Study on the mechanism of retinal nerve protection and intervention for visual impairment | retinal ganglion cells, retinitis pigmentosa, neuroprotection, corneal endothelial cells, retina, optical coherence tomography, angiography, muller cells, visual impairment, cataract surgery, visual perception, visual attention, glaucoma, lens, AMD, photoreceptors, photoreceptor degeneration, diabetes, cornea |
|  | 2-11 | Multi-dimensional study of ocular cytology | electroretinogram, retina, zebrafish, differentiation, muller glia, corneal epithelial cells, diabetic retinopathy, stereopsis, keratocytes, melanopsin, toxic epidermal necrolysis, uveitis, inflammation, depth perception, melatonin, regeneration, nystagmus, cornea |
| 2018-2019 | 3-1 | Mechanism and intervention strategy of oxidative stress and visual impairment in ocular diseases | reactive oxygen species, low vision, diabetic retinopathy, retinitis pigmentosa, oxidative stress, color vision, visual function, erythropoietin, glaucoma, pathogenesis, amniotic membrane, numerosity perception, blepharitis, retina, mesenchymal stem cells, inflammation, visual stability |
|  | 3-2 | Study on the regulatory mechanisms of inflammation, neovascularization and apoptosis in ocular diseases and their effects on visual function | inflammation, neovascularization, apoptosis, oxidative stress, fungal keratitis, eye movements, eye tracking, cornea, diabetic retinopathy, eye movement, muller cell, genetics, substance p, scene perception, angiogenesis, regeneration, binocular rivalry, refractive surgery |
|  | 3-3 | Multimodal evaluation of ocular structure and function and its application and intervention in common ocular diseases | optical coherence tomography, conjunctiva, macular edema, retinal vein occlusion, ocular surface, contact lens, development, cones, visual perception, electrophysiology, diabetic macular edema, vision, stem cells, cornea, retina, cataract, age-related macular degeneration, dry eye disease, endophthalmitis |
|  | 3-4 | Oxidative stress and gene regulatory networks in retinal degenerative diseases | aging, age-related macular degeneration, retinitis pigmentosa, lacrimal gland, dexamethasone, choroidal neovascularization, drusen, transcriptome, rna sequencing, detection, gene expression, oxidative stress, retina, retinal pigment epithelium, angiogenesis, deep learning, visual search |
|  | 3-5 | Studies of myopia and visual impairment: from molecular mechanisms to visual function assessment and intervention strategies | myopia, visual impairment, image processing, migration, homocysteine, refractive error, oxidative stress, exercise, conjunctivitis, ZO-1, phototoxicity, face, collagen, neuroprotection, macula, stereopsis, cone density, multimodal imaging |
|  | 3-6 | Multimodal imaging and molecular mechanism of eye diseases and its application in vision protection and diagnosis | imaging, adaptive optics, keratoconus, rat, optic nerve, corneal endothelial cells, blindness, sjogren's syndrome, visual acuity, spatial vision, pericyte, optic neuropathy, transparency, EEG, rapamycin, electroretinography, exosomes, diagnostic tests |
|  | 3-7 | Study of the global epidemiology, prevention and treatment strategies of eye diseases | epidemiology, trachoma prevalence, trichiasis, global trachoma mapping project, sjogren syndrome, gene therapy, retinopathy of prematurity, photoreceptors, glaucoma, optic flow, metabolic syndrome, public health, visual pathway, prediction, endothelium, neoplasia |
|  | 3-8 | Mechanisms and neuroprotective strategies of retinal cells and their associated signaling pathways in visual perception and ocular diseases | retinal ganglion cells, pupil, face perception, serial dependence, vascular endothelial growth factor, perceptual learning, metabolomics, melanopsin, glaucoma, optic nerve, taurine, retina, optic neuritis, spatial frequency, mouse, macular pigment, age-related macular degeneration, neuroprotection |
|  | 3-9 | Multi-dimensional study of retinal diseases: from neuroprotective mechanisms to diagnostic markers and therapeutic targets | neuroprotection, diabetic retinopathy, microglia, retinal degeneration, uveitis, pterygium, micro-RNA, glaucoma, AMD, photoreceptor, optical coherence tomography, biomarker, uveal melanoma, retinal ganglion cell, VEGF, age-related macular degeneration, muller glia, retinoblastoma |
|  | 3-10 | Study on pathological mechanism and innovative treatment strategy of corneal diseases and diabetic retinopathy | dry eye, drug delivery, proteomics, optical coherence tomography angiography, optical coherence tomography, diabetic retinopathy, inflammation, autophagy, crosslinking, retinal vasculature, macular degeneration, retinal detachment, blood flow, wound healing, corneal neovascularization |
|  | 3-11 | Study of the pathogenesis, diagnosis, treatment of ocular diseases | keratitis, transplantation, glaucoma, motion perception, screening, trauma, melatonin, validation, ocular surface squamous neoplasia, TGF-beta, orbit, questionnaire, POAG, fibrosis, intraocular pressure, vision, impairment, aging, toll-like receptor 4 |
| 2020-2021 | 4-1 | Study on the pathogenesis, diagnosis and intervention strategies of diabetic retinopathy and its complications | retinal detachment, diabetic macular edema, diabetic retinopathy, vitamin D visual impairment, inflammasome, differentiation, vision impairment, depression, retinal organoids, data visualization, NLRP3, natural language processing, high myopia, optical coherence tomography |
|  | 4-2 | Comprehensive study on the relationship between innate immunity and visual system development and function | innate immunity, blindness, cytokines, peripheral vision, crowding, retinal ganglion cell, perception of art, single nucleotide polymorphism, image analysis, object recognition, ganglion cell axon bundles, visual impairment, body perception, bioinformatics, ocular surface, hierarchical processing, toll-like receptor |
|  | 4-3 | Pathogenesis analysis, diagnostic screening, and development of personalized treatment strategies for eye diseases based on machine learning | machine learning, artificial intelligence, deep learning, fibrosis, visual function, optic nerve head, phagocytosis, RNA sequencing, pathogenesis, visual acuity, corneal endothelial cells, treatment, corneal injury, deep convolutional neural networks, vergence, corneal neovascularization, retinal vein occlusion |
|  | 4-4 | Comprehensive study of the effects of Graves' disease and other eye diseases on visual adaptation, holistic perception, and mental health in children | graves' disease, visual adaptation, child health, confidence, genetics, ensemble perception, case report, prediction, olfactory dysfunction, CNGB1, color, NOX4, natural scene statistics, amplitude spectrum, eye banking, cytomegalovirus retinitis, macrophages, aflibercept, biological motion |
|  | 4-5 | Study on the mechanism of exosomes in retinal inflammation, oxidative stress and treatment in uveitis and related ocular diseases | uveitis, exosomes, retinal pigment epithelium, therapy, OCT, inflammation, eye movements, head movements, retinal vasculature, oxidative stress, ocular inflammation, extracellular vesicles, glaucoma, transcriptomics, reactive oxygen species, retinitis pigmentosa, TNF alpha, thiol, mouse |
|  | 4-6 | The regulatory mechanism of autophagy and cell proliferation, plasticity and angiogenesis in anterior segment disease | visual perception, autophagy, dry eye disease, hyaluronan, proliferation, plasticity, primary open-angle glaucoma, neovascularization, bruch's membrane, angiogenesis, fungal keratitis, real-time polymerase chain reaction, face perception, liposomes, corneal epithelial wound healing, HIF-1 alpha |
|  | 4-7 | Mechanisms of interaction of ocular diseases with pathogen infections and environmental factors | cataract, retinal degeneration, eye, aspergillus fumigatus, photoreceptors, signaling pathways, biomarker, ultraviolet radiation b, orbital disease, toll-like receptors, pseudomonas aeruginosa, lens, open angle glaucoma, mouse model, covid-19, oxidative stress |
|  | 4-8 | Meta-analysis of ophthalmic diagnostic techniques on retinopathy | polymorphism, retinopathy, age-related macular degeneration, motion perception, diabetic retinopathy, vitamin D, meta-analysis, regenerative medicine, bibliometrics, optical coherence tomography angiography, biomarkers, toll-like receptor 4, corneal confocal microscopy |
|  | 4-9 | Cytological and epidemiology study in the treatment of eye diseases | mesenchymal stem cells, mice thyroid, eye disease, neoplasia, epidemiology, lacrimal gland, graves' orbitopathy, cell therapy, adipogenesis, retina, sensitivity, perceptual bias, neutrophils, uveitis, keratitis, retinal pigment epithelium |
|  | 4-10 | Mechanisms of retinal ganglion cell protection and optic nerve regeneration driven by virtual reality and eye tracking technology | glaucoma, visual attention, retinal ganglion cells, virtual reality, inflammation, MicroRNA, attention, visual search, transplantation, imaging visual, working memory, eye movements, optic nerve, vascular endothelial growth factor, apoptosis, eye tracking, refractive error, neuroprotection |
| 2022-2023 | 5-1 | Multi-dimensional study on the pathological mechanism and visual perception of ocular diseases based on optical coherence tomography | optical coherence tomography, orbit, retina, pathology, visual perception, inflammation, imaging, macula, retinal pigment epithelium, ocular, optic nerve head, fibrosis, perception, prevalence, genetics, neoplasia, anatomy, retinal dystrophy, cataract |
|  | 5-2 | Epidemiological studies and public health responses to the impact of COVID-19 on eye health | epidemiology, public health, covid-19, diagnostic tests, retina, dry eye, imaging, glaucoma, treatment surgery, patient education, infection, mucormycosis, wound healing, lacrimal gland, readability, vision, cataract surgery, psychophysics |
|  | 5-3 | Multi-dimensional study of ophthalmic diseases: Innovative exploration from pathological mechanism to clinical diagnosis and treatment | dry eye disease, endophthalmitis, neurodegeneration, optical coherence tomography, angiography, binocular vision, foveal avascular zone, glaucoma, myopia, visual search, mitochondria, diabetic retinopathy, choroidal neovascularization, apoptosis, melanoma, microbial keratitis, metabolomics |
|  | 5-4 | Comprehensive study on the pathological mechanism, visual function and drug intervention of glaucoma | glaucoma, accommodation, NLRP3, inflammation, VEGF, contrast sensitivity, hypertension, intraocular pressure, neovascularization, drugs, visual function, pharmacology, social determinants of health |
|  | 5-5 | Comprehensive study of diabetic retinopathy, visual function and related risk factors | diabetic retinopathy, gaze, age-related macular degeneration, retinal thickness, retina, diagnosis, conjunctiva, optical coherence tomography, risk factors, vitreous, attention, clinical trial, extracellular matrix, reactive oxygen species, ocular surface |
|  | 5-6 | Comprehensive study of eye disease diagnosis and vision protection based on artificial intelligence | optic nerve, artificial intelligence, visual acuity, depression, diabetic retinopathy, machine learning, retinopathy of prematurity, retinoblastoma, vision impairment, deep learning, retinitis pigmentosa, microbiology, age-related macular degeneration, refractive surgery |
|  | 5-7 | Multi-dimensional study of eye disease: pathological mechanisms and intervention strategies | angiogenesis, microglia, glaucoma, uveitis, pain, cornea, refractive error, fibrosis, smoking, MRSA, lens, cataract, corneal epithelium, biomechanics, stem cells, contact lenses |
|  | 5-8 | Multi-omics study of COVID-19-associated retinal microenvironmental alterations and inflammatory mechanisms | covid-19, sars-cov-2, proteomics, sarcoidosis, retina/ uveitis, aqueous humor, inflammation, age-related macular degeneration, eye, cytokines, anti-vegf, immunohistochemistry ,ophthalmology /corticosteroid, apoptosis, youtube |
|  | 5-9 | Study on pathogenesis, biomarkers and treatment strategies of diabetic retinopathy and related eye diseases | biomarker, oxidative stress, inflammation, OCTA, pathogenesis, age-related macular degeneration, diabetic retinopathy, bevacizumab, hypoxia, eye (globe), fungal keratitis, intravitreal injection, genetics, neuroprotection, retinal blood flow, electroretinogram |
